# Deficiency for MicroRNA-582 does not impact dilated cardiomyopathy or heart failure induced by pressure overload *in vivo*

**DOI:** 10.3389/fcell.2025.1661965

**Published:** 2025-11-10

**Authors:** Simone Martini, Inka Dobberstein, Nesrin Schmiedel, Lucia Sophie Kilian, Jakob Christoph Voran, Frauke Senger, Ashraf Yusuf Rangrez, Derk Frank, Christian Kuhn, Norbert Frey

**Affiliations:** 1 Department of Internal Medicine III, Cardiology and Critical Care, University Hospital Schleswig-Holstein, Kiel, Germany; 2 German Centre for Cardiovascular Research (DZHK), Partner Site Hamburg/Kiel/Lübeck, Kiel, Germany; 3 Roche Diagnostics GmbH, Mannheim, Germany; 4 IUF – Leibniz-Institut für umweltmedizinische Forschung GmbH, Düsseldorf, Germany; 5 Department of Internal Medicine III, Cardiology, University of Heidelberg, Heidelberg, Germany; 6 German Centre for Cardiovascular Research (DZHK), Partner Site Heidelberg/Mannheim, Heidelberg, Germany; 7 Department of Cardiology, Critical Care and Angiology, ViDia Christliche Kliniken Karlsruhe, Karlsruhe, Germany

**Keywords:** miR-582-5p, MicroRNAs, heart failure, cardiomyopathy, cardiac hypertrophy, Transverse aortic constriction (TAC), Calsarcin-1

## Abstract

MicroRNAs (miRNAs) are critical post-transcriptional regulators of gene expression and have been extensively implicated in cardiovascular development, homeostasis, and disease. Among them, microRNA-582 (miR-582) has been associated with several non-cardiac pathologies, yet its role in the heart remains poorly characterized despite significant cardiac expression. In this study, we investigated the functional significance of miR-582 in cardiac pathophysiology through both gain- and loss-of-function approaches. We observed differential expression of miR-582 in murine models of cardiomyopathy, prompting further mechanistic evaluation. Thus, we generated transgenic mice with cardiac-specific overexpression of miR-582 (TG-582) as well as miR-582 knockout (582-KO) mice. Neither model exhibited an obvious cardiac phenotype under basal conditions. Following pressure overload via transverse aortic constriction (TAC), both TG-582 and 582-KO mice developed hypertrophy and functional adaptations comparable to wildtype controls. Additionally, crossbreeding these models with Calsarcin-1-knockout (CS1-KO) mice, a model of dilated cardiomyopathy, did not modify the pathological phenotype. These results indicate that miR-582 does not play a determinative role in pressure overload-induced cardiac hypertrophy or in the progression of dilated cardiomyopathy. Our findings highlight the importance of rigorously controlled *in vivo* studies to accurately define the cardiac miRNA landscape and to guide future therapeutic strategies.

## 1 Introduction

Heart failure is a complex and life-threatening condition associated with significant morbidity and mortality. With over 56 million individuals affected globally, substantial efforts are being made to mitigate its societal and economic burden (Khan et al., 2024). A detailed understanding of the molecular mechanisms underlying heart failure development and progression is essential for improving current treatment strategies and identifying novel therapeutic targets. Among the primary contributors to heart failure are cardiac hypertrophy due to pressure overload and dilated cardiomyopathies, underscoring the importance of uncovering their pathophysiological mechanisms to facilitate therapeutic innovation (Frey et al., 2004a; Gigli et al., 2024; Nakamura and Sadoshima, 2018).

MicroRNAs are key regulators of a variety of biological processes, including metabolism, proliferation, differentiation, apoptosis, calcium handling, and fibrosis. They have been implicated in the pathogenesis of numerous diseases, including cardiovascular disorders (Nemeth et al., 2024). Several microRNAs have been identified as modulators of cardiac hypertrophy and heart failure, raising the possibility of therapeutic intervention through targeted modulation of their expression (Laggerbauer and Engelhardt, 2022). Clinical trials evaluating microRNA-based therapies, including cardiac-specific interventions, have been completed or are ongoing (Iacomino, 2023). However, a substantial number of microRNAs remain uncharacterized, particularly in the context of cardiovascular disease.

MicroRNA-582 (miR-582), while relatively underexplored in cardiac biology, has been implicated in tumor suppression across multiple cancer types (Uchino et al., 2013; Wang et al., 2020; Xiao et al., 2022; Zhang et al., 2015) and associated with non-cardiac muscle disorders such as Facioscapulohumeral muscular dystrophy (FSHD) (Portilho et al., 2015) and osteoarthritis (Diaz-Prado et al., 2012). In vascular tissue, miR-582 has been shown to promote fibrotic remodeling within the carotid arteries of aged rats and in response to simulated microgravity (Zhang et al., 2024). Within the cardiovascular context, preliminary studies have suggested a role for miR-582-5p in ischemic cardiomyocyte injury, potentially mediated through direct targeting of cAMP response element-binding protein 1 (*Creb1*) (Niu et al., 2022) and Coagulation Factor II Thrombin Receptor-Like 2 (*F2rl2*) (Wu et al., 2022). Nonetheless, the broader contribution of miR-582 to cardiac pathophysiology remains incompletely defined.

In light of our own transcriptomic analyses revealing consistent dysregulation of miR-582 across multiple *in vitro* and *in vivo* cardiomyopathy models, we sought to systematically investigate its functional relevance in both cardiac hypertrophy and dilated cardiomyopathies, two major drivers of heart failure. To this end, we employed two well-established *in vivo* experimental systems: pressure overload via transverse aortic constriction (TAC) (Bosch et al., 2021; Rockman et al., 1991), and the Calsarcin-1-knockout (CS1-KO) mouse model of dilated cardiomyopathy (Frey et al., 2004b; Osio et al., 2007; Rangrez et al., 2017; Schoensiegel et al., 2007). We generated and phenotypically characterized two complementary models, transgenic mice with cardiac-specific miR-582 overexpression (TG-582), and miR-582 knockout (582-KO) mice. Neither model displayed a baseline cardiac phenotype. Furthermore, miR-582 modulation did not affect the cardiac remodeling response to either TAC-induced pressure overload or genetic cardiomyopathy in the CS1-KO background.

Taken together, our findings do not support a critical role for miR-582 in the pathogenesis of hypertrophic or dilated cardiomyopathy. These data suggest that therapeutic targeting of miR-582 is unlikely to yield benefit in these settings and underscore the importance of rigorously evaluating candidate miRNAs within relevant pathophysiological models *in vivo*.

## 2 Materials and methods

### 2.1 Isolation and culture of neonatal rat cardiac myocytes

For neonatal rat ventricular cardiac myocytes (NRVCM) preparation, female and male Wistar rats one to 3 days of age were sacrificed by decapitation. Hearts were extracted and placed in ADS buffer (120 mmol/L NaCl, 20 mmol/L HEPES, 9 mmol/L NaH2PO4 x H2O, 6 mmol/L glucose, 5 mmol/L KCl, 0.8 mmol/L MgSO4 x 7H2O (pH 7.4)). Ventricles were isolated, minced and digested in collagenase type II (0.5 mg/mL, Worthington Biochemical Corporation) and pancreatin (0.6 mg/mL, Sigma-Aldrich) in sterile ADS buffer at 37 °C in four to five steps. The cell suspension was divided using a Percoll (GE Healthcare) gradient to separate cardiomyocytes from fibroblasts. Cardiomyocytes were plated in DMEM supplemented with 10% (v/v) FCS, 100 U/mL penicillin and 100 μg/mL streptomycin (Thermo Fisher Scientific), and 2 mM L-glutamine (Thermo Fisher Scientific) for 24 h and subsequently infected with adenoviral vectors in serum free medium. After 48 h the medium was replaced and cells were stimulated with 100 μM phenylephrine or 100 nM endothelin-1 (Sigma-Aldrich) or subjected to bidirectional cyclic stretch (FlexCell Systems) for 48 h.

### 2.2 Animal experiments

All experiments involving animals were approved by the Ministry of Agriculture, Rural Areas, European Affairs and Consumer Protection of Schleswig-Holstein (Germany) and were performed according to EU Directive 2010/63/EU and ARRIVE-guidelines. Mice aged 8–12 weeks were used for all experiments.

Calcineurin-TG mice (C57BL/6-Tg(α-MHC-Ppp3ca)37Eno/0) express a constitutively active calcineurin under the α-MHC promotor in the heart (Molkentin et al., 1998). *CS1*-KO mice carry two disrupted nonfunctional Myozenin2 alleles (Frey et al., 2004b; Rangrez et al., 2017; Schoensiegel et al., 2007). Muscle LIM protein (*MLP*)-knockout mice carry two disrupted nonfunctional Csrp3 alleles (Arber et al., 1997).

#### 2.2.1 Generation of TG-582 and miR-582-knockout mice

MiR-582-knockout mice (B6N.JM8A-MiR582tm1Kuv, RRID: MMRRC_036383-UCD, Background: C57BL/6N) were purchased from MMRRC (UC Davis, United States). The microRNA sequence was replaced via homologous recombination by a LoxP-F3-PGK-EM7-PuroΔtk-bpA-LoxP-FRT targeting vector containing 5.5 kb (5′) and 3.3 kb (3′) arms of homology. The EM7/PGK promoter puroDtk cassette was removed by Cre transfection followed by FIAU selection to create a “clean” null allele (Prosser et al., 2011). Transgenic TG-582 mice (C57BL/6-Tg(aMHC-582)3Kuv/N) were generated using a pBS-SKII + αMHC FLAG hGH1 vector and carry randomly integrated constructs (pre-miR-582 + 110 bp 5’/+150 bp 3′) expressing mmu-miR-582 under an α-MHC promotor with an hGH1 polyA signal.

#### 2.2.2 Transverse aortic constriction (TAC)

Before TAC surgery the animals received analgesia (buprenorphine 0.1 mg/kg body weight) subcutaneously and were anesthetized with isoflurane. During the procedure mice were orally intubated and ventilated at 120–130 breaths per minute (200 µL tidal volume). After lateral thoracotomy through the second intercostal space, the transverse aorta was ligated between the brachiocephalic and left carotid artery against a 27-gauge needle as spacer. After removing the needle, the chest was closed. Sham animals underwent identical surgery without ligation and served as a control. Mice were killed by cervical dislocation 14 days post TAC and cardiac tissue was collected. The animals were randomly assigned to the experimental groups.

#### 2.2.3 Echocardiography

Echocardiography was performed on anaesthetized mice with isoflurane (1%–4% v/v) on a Vevo 1,100 ultrasound system (VisualSonics, FUJIFILM, Japan) or Vivid 7 Pro Ultrasound System (GE healthcare). During the procedure mouse body temperature was maintained at 37 °C and the heart rate was controlled continuously. After echocardiography mice were killed by cervical dislocation.

### 2.3 RNA isolation, reverse transcription, and quantitative real-time PCR

Total RNA was isolated from NRVCM and from cardiac tissue (left ventricle (LV)) using the QIAzol kit (Qiagen). After DNase I (Thermo Fisher Scientific) digestion RNA concentration was measured with Nanodrop spectrophotometer (Thermo Fisher Scientific). cDNA was synthesized from 1 μg RNA using a hexanucleotide random-primer-mix (Carl Roth) and the SuperScript® III First-Strand Synthesis System (Thermo Fisher Scientific) or the miScript II RT kit (Qiagen). For Quantitative real-time PCR (qRT-PCR) 10 ng cDNA was used with miScript Primer Assays and QuantiTect SYBR Green Master Mix (Qiagen) or BioRad iQ Multiplex Powermix for MultiplexPCR in a CFX96 real-time PCR detection system (Bio-Rad). Rpl32, 18S, U6 or Snord25 were used for normalization. Primer and probe sequences see Supplementary Table S2.

### 2.4 Histological staining

For cryo-sections, a slice from the LV was placed in a mould with embedding compound (Tissue-Tek O.C.T. Compound, Sakura), snap-frozen on dry ice and stored at −80 °C. For staining 5 µm thick serial sections were used. After staining sections were mounted with coverslips using xylene-soluble mounting medium (Cytoseal Mountant XYL, Fisher Scientific). Haematoxylin-Eosin (HE) staining was performed by placing slides in haematoxylin solution (Weigert’s Iron Haematoxylin, Sigma-Aldrich) for 5 min, washed tap water for 5 min, then placed in eosin solution (Eosin G in 95% ethanol) for 2 min and then washed by dipping in distilled water. Subsequently the slides were dipped in 50%, 70%, 95% and 100% ethanol and xylene. Fast green/Sirius red staining was performed using a 0.5% Fast green (Fast Green FCF dye, Sigma-Aldrich) and 0.1% Sirius red (Sirius red F3BA dye, Sigma-Aldrich) dissolved in 1.2% picric acid solution (BioChemica). Slides were placed in the staining solution for 4 days, quickly washed with tap water, then dipped in 70%, 96% and 100% ethanol and xylene.

### 2.5 Target gene prediction

Putative targets were predicted using four algorithms: MicroCosm (miRanda 3.0), TargetScanMouse v7.0, miRDB (MirTarget) and DIANA-microT-CDS. Only genes predicted by all four tools were considered for downstream analysis.

### 2.6 Statistical analysis

All data are presented as the mean ± standard error of the mean (SEM), unless stated otherwise. Statistical comparisons between groups were performed using unpaired two-tailed Student’s t-test, one-way-ANOVA or two-way-ANOVA followed by Tukey’s *post hoc* test in the software GraphPad Prism 8.4.3. P < 0.05 was considered to be statistically significant ( *p < 0.05, **p < 0.01, ***p < 0.005, ****p < 0.001).

## 3 Results

### 3.1 MiR-582 expression is differentially regulated in several models of cardiac hypertrophy

**FIGURE 1:**
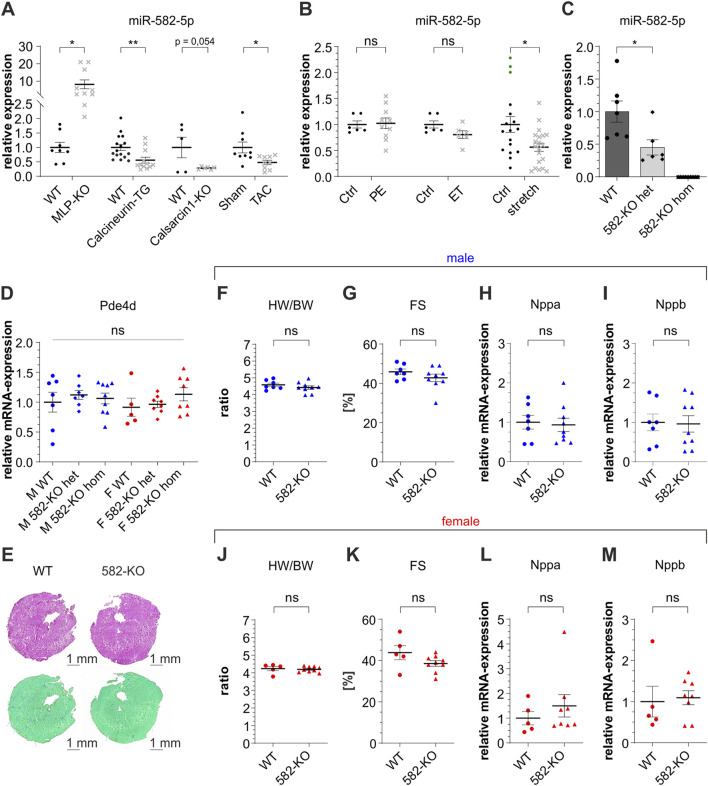
Expression of miR-582-5p and basic characterization of 582-KO mice. Relative expression of miR-582-5p in different *in vivo* (**(A)**, mouse) and *in vitro* (**(B)**, NRVCM) models of cardiomyopathy, n = 5-16 (mice), n = 2-4 experiments (NRVCM), **(C)** relative expression of miR-582-5p in heterozygous and homozygous 582-KO mice, **(D)** RNA-expression of Pde4d in male (blue) and female (red) mice, **(E)** Hematoxylin-eosin staining (top) and Sirius Red/Fast Green Collagen Staining (bottom), **(F–I)** morphometric and echocardiographic data and expression of *Nppa* and *Nppb* in male mice, **(J–M)** morphometric and echocardiographic data and expression of *Nppa* and *Nppb* in female mice, n = 5-9 animals, (BW, body weight; Ctrl, control; ET, endothelin 1; F, female; FS, fractional shortening; het, heterozygous; hom, homozygous; HW, heart weight; KO, knockout; M, male; PE, phenylephrine; WT, wildtype, *p < 0.05, **p < 0.01, ***p < 0.005, ns, not significant).

MiR-582 has gained attention for its involvement in multiple pathophysiological conditions, recently also in the cardiovascular system. To investigate the potential role of miR-582-5p in cardiac hypertrophy and cardiomyopathy, we analyzed its expression in left ventricular tissue across several established *in vivo* models. Notably, miR-582-5p expression was downregulated in *Calcineurin*-transgenic (Molkentin et al., 1998) and *CS1*-KO mice (Frey et al., 2004b; Rangrez et al., 2017; Schoensiegel et al., 2007), but upregulated in *MLP*-KO (Arber et al., 1997) mice, all well-established genetic models of cardiomyopathy and heart failure (Figure 1A). Additionally, TAC, an experimental model inducing pressure overload, resulted in a significant reduction of miR-582-5p expression in murine cardiac tissue (Figure 1A).

To extend these findings, we examined miR-582-5p regulation in neonatal rat ventricular cardiomyocytes (NRVCM) subjected to different hypertrophic stimuli. Treatment with phenylephrine (PE) or endothelin-1 (ET), agents known to elevate intracellular calcium and induce hypertrophic growth, did not significantly alter miR-582-5p expression (Figure 1B). In contrast, mechanical stretch, simulating biomechanical overload, led to a decrease in miR-582-5p levels (Figure 1B).

In summary, these findings demonstrate that miR-582-5p is differentially regulated in several *in vivo* and *in vitro* models of cardiac hypertrophy, suggesting a context-dependent role in myocardial stress responses.

To elucidate the physiological role of miR-582 in cardiac function *in vivo*, we generated two genetically modified murine models: a transgenic line with cardiac-specific overexpression of miR-582 driven by a α-myosin heavy chain (αMHC) promoter (TG-582), and a knockout line with global deletion of miR-582 (582-KO). In the following, we first report the results obtained in the KO model, followed by those from the transgenic line.

### 3.2 582-KO mice do not reveal a cardiac phenotype

Quantitative PCR analysis confirmed a reduction of miR-582-5p expression in the heart of heterozygous 582-KO mice and a complete absence in homozygous counterparts (Figure 1C). Notably, miR-582 is located within an intronic region of its host gene phosphodiesterase 4d (*Pde4d*) gene; however, Pde4d expression levels in cardiac tissue remained unaltered in 582-KO mice (Figure 1D).

Histological analyses, including collagen- and hematoxylin-eosin staining did not reveal any discernible differences in cardiac morphology between 582-KO and WT mice (Figure 1E). Furthermore, morphometric assessments, conducted in male and female mouse cohorts, including heart weight-to-body weight ratios in 8- to 10-week-old mice, revealed no significant differences between 582-KO and wildtype (WT) littermates (Figures 1F,J). Echocardiographic evaluations demonstrated preserved cardiac function, with fractional shortening measurements indicating normal baseline contractility across both groups (Figures 1G,K). Additionally, expression levels of natriuretic peptide A (*Nppa)* and natriuretic peptide B (*Nppb)*, established markers of cardiac hypertrophy and heart failure, were comparable between WT and 582-KO mice (Figures 1H,I,L,M). These observations were consistent across both male (blue) and female (red) cohorts.

Collectively, these findings suggest that deletion of miR-582 does not significantly impact baseline cardiac morphology or function in murine models.

### 3.3 582-KO mice display no cardiac phenotype after induced pressure overload

Based on the differential regulation under pathophysiological conditions and the unremarkable baseline characterization of the 582-KO mouse, we hypothesized that its function only becomes evident under stress conditions.

**FIGURE 2:**
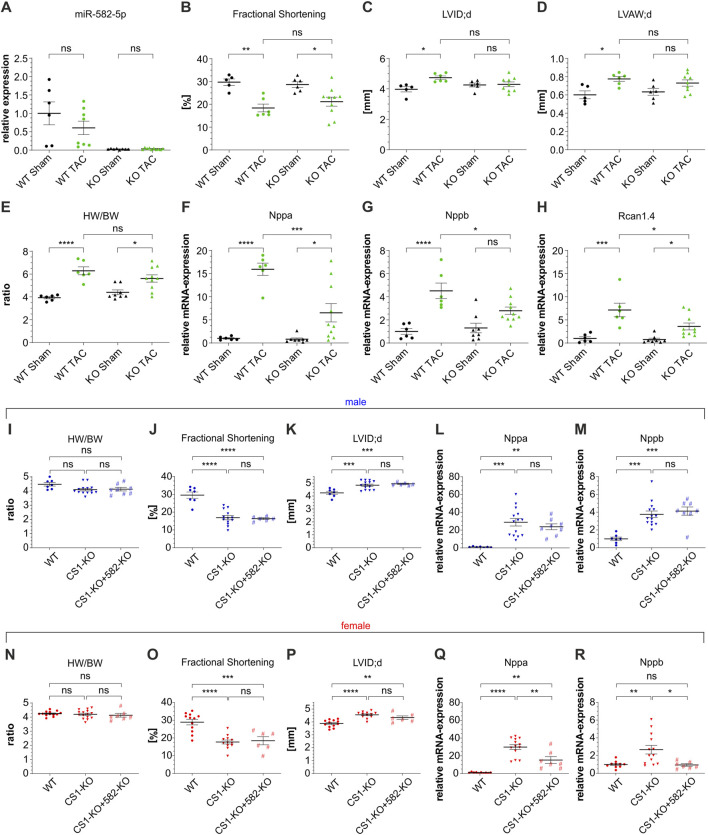
TAC surgery in male 582-KO mice **(A–H)** and crossbreeding *CS1*-KO/582-KO mice **(I-R)**. **(A)** relative expression of miR-582-5p, **(B–D)** echocardiographic data, **(E)** Heart weight/body weight ratio, **(F–H)** expression of *Nppa, Nppb* and *Rcan1.4* (n = 5-10 animals), **(I–R)** morphometric and echocardiographic data and expression of *Nppa* and *Nppb* of male **(I–M)** and female **(N–R)** *CS1*-KO/582-KO cross bred mice, n = 6-15 animals (BW, body weight; *CS1*, Calsarcin-1; HW, heart weight; KO, knockout; LVAW; d, left ventricular anterior wall thickness at end-diastole; LVID; d, left ventricular internal diameter at end diastole; TAC, transverse aortic constriction; WT, wildtype, *p < 0.05, **p < 0.01, ***p < 0.005, ****p < 0.001, ns, not significant).

To assess the role of miR-582 under such stress conditions, we subjected 8-week-old male 582-KO and WT mice to transverse aortic constriction (TAC). TAC is a well-established murine model for inducing pressure overload, leading to cardiac hypertrophy and eventual heart failure. Two weeks post-surgery, comprehensive analyses—including morphometric assessments, echocardiography, and evaluation of hypertrophic gene markers—were conducted. MiR-582-5p expression was reduced to background levels in 582-KO mice (Figure 2A). As anticipated, WT mice developed significant hypertrophy following TAC, evidenced by reduced cardiac function, enhanced ventricular internal diameter and wall thickness, increased heart weight-to-body weight ratios and elevated expression of hypertrophic markers such as *Nppa*, *Nppb* and regulator of calcineurin 1 (*Rcan1.4*) (Figures 2B–H). Sham operated 582-KO mice exhibited parameters comparable to their WT counterparts (Figures 2B–H). Importantly, no significant differences were observed between WT and 582-KO mice in response to pressure overload concerning morphometric and echocardiographic parameters (Figures 2B–E). However, 582-KO mice displayed a significant reduction in the expression of hypertrophic marker genes post-TAC compared to WT mice (Figures 2F–H). These findings suggest that while miR-582 deletion does not markedly alter structural and functional cardiac responses to pressure overload, it may differentially influence molecular hypertrophic signaling pathways.

### 3.4 MiR-582 deficiency does not alter calsarcin-1-knockout cardiac phenotype

*CS1*–deficient mice on a pure C57BL/6 background as used in this study develop dilated cardiomyopathy characterized by contractile dysfunction and upregulation of fetal hypertrophic genes (Rangrez et al., 2017). To assess whether the observed downregulation of miR-582 in these animals contributes to the *CS1*-KO cardiac phenotype, we generated *CS1*-KO/582-KO double-knockout mice and evaluated baseline cardiac parameters. Echocardiographic assessments and gene expression analyses of male (blue) and female (red) *CS1*-KO mice confirmed reduced cardiac function and elevated expression of hypertrophic markers, such as *Nppa* and *Nppb*, consistent with previous findings (Figures 2I–R). Crossbreeding with 582-KO mice did not result in significant alterations in echocardiographic or morphometric parameters compared to *CS1*-KO mice in either gender (Figures 2I–K,N–P). However, a nonsignificant trend toward attenuation of the induction of hypertrophic gene markers was observed in female double-knockout mice (Figures 2Q,R), whereas no differences were detected in male mice (Figures 2L,M).

### 3.5 TG-582 mice do not show a baseline cardiac phenotype

**FIGURE 3:**
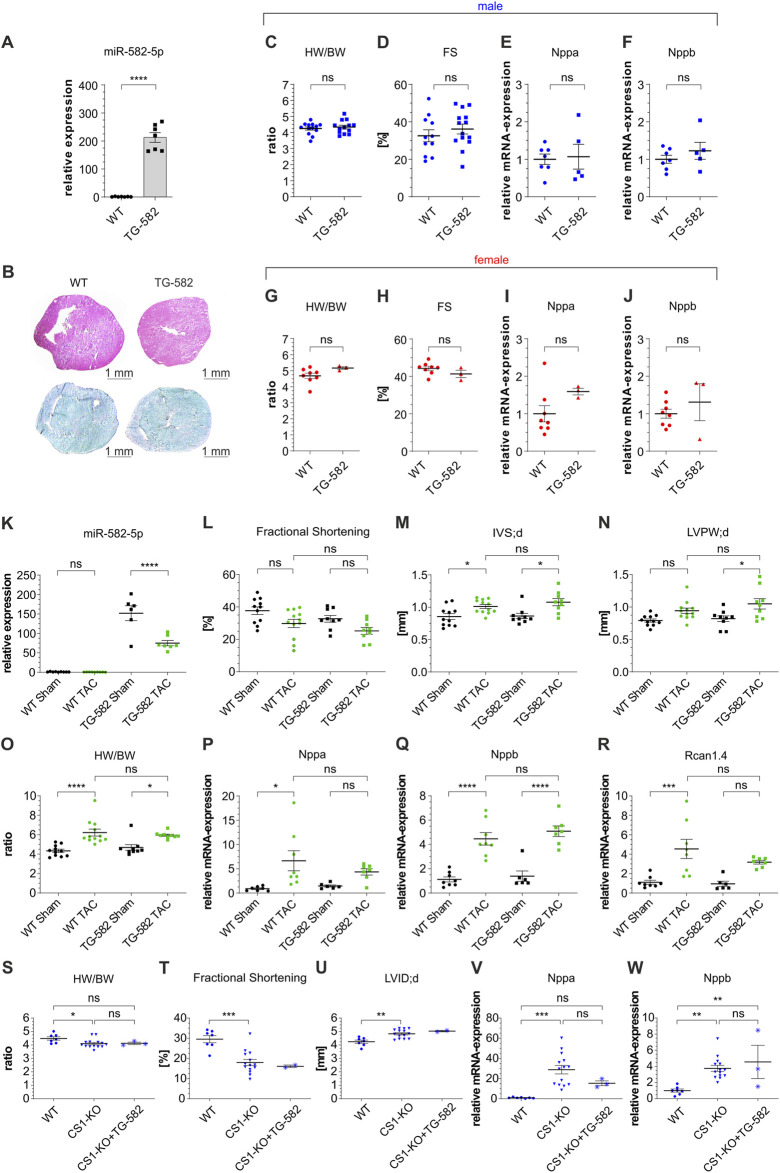
Basic characterization of TG-582 mice **(A–J)**, TAC surgery in male TG-582 mice **(K–R)** and crossbreeding of male *CS1*-KO/TG-582 mice **(S-W)**. **(A)** relative expression of miR-582-5p, **(B)** Hematoxylin-eosin staining (top) and Sirius Red/Fast Green Collagen Staining (bottom), **(C–F)** morphometric and echocardiographic data and expression of *Nppa* and *Nppb* in male mice, **(G–J)** morphometric and echocardiographic data and expression of *Nppa* and *Nppb* in female mice, n = 3-14 animals; **(K)** relative expression of miR-582-5p, **(L–N)** echocardiographic data, **(O)** Heart weight/body weight ratio, **(P–R)** expression of *Nppa, Nppb* and *Rcan1.4*, n = 6-12 animals; **(S–W)** morphometric and echocardiographic data and expression of hypertrophic marker genes in male *CS1*-KO/TG-582 cross bred mice, n = 2-14 animals, (BW, body weight; *CS1*, Calsarcin-1; FS, fractional shortening; HW, heart weight; IVS; d, interventricular septal thickness at end-diastole; KO, knockout; LVID; d, left ventricular internal diameter at end diastole; LVPW; d, diastolic left ventricular posterior wall thickness at end-diastole; TAC, transverse aortic constriction; WT, wildtype, *p < 0.05, **p < 0.01, ***p < 0.005, ****p < 0.001, ns, not significant).

We next examined the cardiac effects of miR-582 overexpression in TG-582 mice. Quantitative analysis confirmed a significant upregulation of miR-582-5p in the hearts of TG-582 mice compared to WT controls (Figure 3A). Histological analyses, including collagen staining for fibrosis and hematoxylin-eosin staining for overall myocardial architecture, demonstrated no apparent differences in cardiac morphology between TG-582 and WT mice (Figure 3B). Comprehensive morphometric assessments, including heart weight-to-body weight ratios, revealed no significant differences between TG-582 and WT mice at 8 weeks of age (Figures 3C,G). Echocardiographic evaluations demonstrated preserved baseline cardiac function, with fractional shortening comparable between both groups (Figures 3D,H). Likewise, the expression levels of hypertrophic markers *Nppa* and *Nppb* remained unchanged in TG-582 mice relative to WT counterparts (Figures 3E,F,I,J). These observations were consistent across both male (blue) and female (red) cohorts, aligning with observations from 582-KO mice.

### 3.6 TG-582 mice display no cardiac phenotype after induced pressure overload

To investigate the impact of miR-582 overexpression under pathological stress, male TG-582 mice were subjected to TAC to induce pressure overload. Assessment of miR-582-5p expression showed a substantial increase in TG-582 mice compared to wildtypes and a significant decrease after TAC in transgenic mice compared to sham operated mice (Figure 3K). Post-TAC analysis revealed no significant differences between TG-582 and WT mice in terms of echocardiographic and morphometric parameters (Figures 3L–O). Furthermore, the expression levels of hypertrophic markers *Nppa, Nppb* and *Rcan1.4* were similarly elevated in both groups following TAC surgery, indicating a comparable hypertrophic response (Figures 3P–R).

### 3.7 Overexpression of miR-582 does not alter dilated cardiomyopathy in calsarcin-1-knockout mice

Complementary to the above investigated effect of miR-582 depletion on the *CS1*-KO cardiac phenotype, we assessed whether upregulation of miR-582 influences the pathological cardiac remodeling observed in *CS1*-KO mice. For this purpose, we crossbred *CS1*-KO mice with TG-582 mice and analyzed basic cardiac parameters. Interestingly, the crossbreeding yielded only three male *CS1*-KO/TG-582 mice and no female counterparts, while breeding numbers for other genotypes remained within normal ranges. Despite the limited sample size, no significant differences were observed between *CS1*-KO and *CS1*-KO/TG-582 male mice across the analyzed parameters (Figures 3S–W).

### 3.8 *Creb1* and *F2rl2* expression remains unaltered in 582-KO or TG-582 mice

**FIGURE 4:**
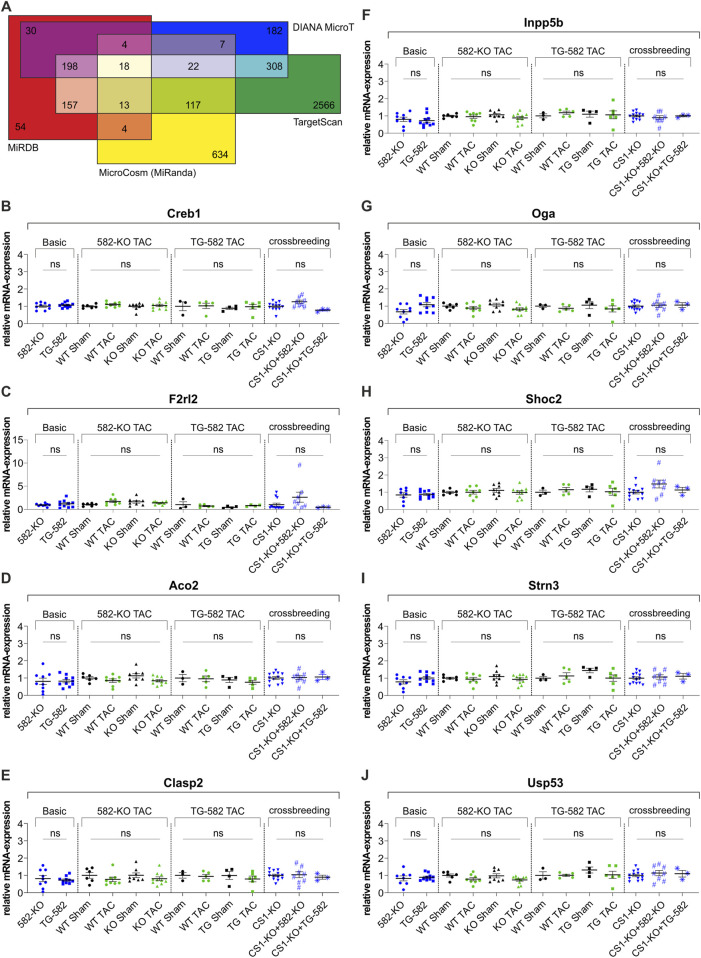
*In silico* target prediction **(A)** and relative RNA-expression of published **(B,C)** and predicted **(D–J)** target genes of miR-582-5p. **(A)** *In silico* target prediction employing the algorithms: MicrocCosm (miRanda), TargetScan, MiRDB (MirTarget) and DIANA-MicroT yielding 18 overlapping predicted target genes, **(B–J)** relative RNA-expression of previously identified miR-582-5p target genes Creb1 **(B)** and F2rl2 **(C)** and relative RNA-expression of seven predicted miR-582-5p target genes **(D–J)** in 582-KO and TG-582 mice, 582-KO and TG-582 mice after TAC-surgery and in *CS1*-KO mice crossbred with 582-KO and TG-582 mice respectively, n = 3-13. (CS1, Calsarcin-1; KO, knockout; TAC, transverse aortic constriction; WT, wildtype; ns, not significant).

In addition to the comprehensive cardiac phenotyping, we examined the expression of cAMP response element-binding protein (*Creb1*) and Coagulation factor II thrombin receptor like 2 (*F2rl2*) — genes previously identified as miR-582 target genes in the heart—in both 582-KO and TG-582 models. Quantitative analyses revealed that cardiac expression levels of *Creb1*- (Figure 4B) and *F2rl2*-mRNA (Figure 4C) did not change in mice, neither under basal conditions, nor following pressure overload induced by TAC or in *CS1*-KO mice when crossed with either experimental model.

Additionally, we performed an *in silico* target prediction to identify putative targets of miR-582-5p, employing four algorithms: MicrocCosm (miRanda) (Enright et al., 2003), TargetScan (Agarwal et al., 2015), miRDB (MirTarget) (Wong and Wang, 2015), and DIANA-microT (Paraskevopoulou et al., 2013) (Supplementary Table S1). This analysis yielded 18 overlapping predicted target genes, of which seven were selected for further investigation based on their potential relevance to cardiac biology (Figure 4A). Transcript levels of these genes were analyzed in cardiac tissue from both 582-KO and TG-582 mice under basal conditions, following pressure overload induced by TAC and in the respective crossbred models. None of the selected targets exhibited significant changes in expression under any of the tested conditions (Figures 4D–J).

These consistent findings across multiple experimental conditions indicate that the modulation of miR-582 does not influence the transcriptional regulation of either of the investigated targets in the analyzed *in vivo* settings.

## 4 Discussion

In the present study, we demonstrated that miR-582-5p is differentially regulated across multiple *in vivo* and *in vitro* models of cardiomyopathy. This finding prompted an in‐depth investigation of its *in vivo* role in dilated cardiomyopathy and pressure overload–induced cardiac hypertrophy with subsequent heart failure. To address this, we generated two novel mouse models: one with a global deletion of miR-582 and another with cardiomyocyte‐specific overexpression of miR-582. Under basal conditions, neither male nor female mice from either model exhibited an overt cardiac phenotype. We then subjected these models to pressure overload via TAC and crossed them with the *CS1*-KO dilated cardiomyopathy model to uncover potential stress‐dependent effects of miR-582. Following TAC, knockout and transgenic mice largely mirrored WT animals, with the only notable difference being a modest attenuation in the upregulation of hypertrophic marker genes in 582-KO mice. One potential limitation is that FS in WT controls declined only marginally post-TAC in the TG-582 model, possibly limiting sensitivity to detect functional differences. In line with the overall findings, crossing 582-KO with *CS1*-KO mice did not alter the *CS1*-KO phenotype, except for a partial suppression of hypertrophic gene induction exclusively in females. It is also noteworthy that the crossbreeding of *CS1*-KO with TG-582 mice produced very few *CS1*-KO/TG-582 offspring—with an absence of females—although surviving animals did not display any overt phenotype.

Recently, two studies described a potential role for miR-582 in ischemic cardiac myocytes and a regulation of miR-582 in myocardial infarction (MI) in mice (Niu et al., 2022; Wu et al., 2022), supporting the notion that miR-582 may have a function in the heart under other pathophysiological conditions. Wu et al. reported that downregulation of the long non-coding RNA (lncRNA) nuclear paraspeckle assembly transcript 1 (NEAT1) attenuates MI of mice via the miR-582-5p/Coagulation factor II thrombin receptor like 2 (F2RL2) axis (Wu et al., 2022). In their work, MI was associated with reduced miR-582-5p levels and concomitant upregulation of F2RL2 (also known as PAR-3). They also demonstrated that downregulation of F2RL2 has a beneficial effect on the outcome of MI in mice and that miR-582-5p negatively regulates F2RL2 *in vitro*, suggesting that hypothetically overexpression of miR-582-5p would lead to an improvement of the effects of MI. However, the potential benefit of miR-582-5p overexpression in MI *in vivo* remains untested. To date, the role of PAR-3 in hypertrophic or dilated cardiomyopathy has not been elucidated, despite evidence that inhibition of PAR-1 and PAR-2 — known interactors of PAR-3 — suppresses cardiac hypertrophy induced by pressure overload and in renin-overexpressing hypertensive mice (Fletcher et al., 2023; Narita et al., 2021; Yokono et al., 2020).

Conversely, Niu et al. (2022) observed an upregulation of miR-582-5p in both MI‐mice and hypoxia/reperfusion‐treated cardiomyocytes, a finding that directly contrasts with Wu et al.’s report. Niu and colleagues identified *Creb1* as a target of miR-582-5p in HL-1 cells, a cardiomyocyte cell line. CREB1, which requires phosphorylation at Ser-133 for activation and subsequent target gene regulation, has been linked to cardiac dysfunction; overexpression of a dominant-negative CREB mutant in cardiomyocytes led to dilated cardiomyopathy with increased mortality (Fentzke et al., 1998), whereas cardiomyocyte-specific CREB deletion in mice yielded a phenotype similar to WT mice (Matus et al., 2007). These discrepant outcomes may be attributable to the dominant-negative mutant’s inability to undergo Ser-133 phosphorylation, a necessary step for CREB-dependent activation of target gene transcription. However, it still retains the capacity to dimerize with other CREB/CREM family members, which hinders their binding to the cAMP response element (CRE) (Fentzke et al., 1998; Mayr and Montminy, 2001). This raises the possibility that the intrinsic role of CREB in the regulation of the development of hypertrophic or dilated cardiomyopathy might be limited (Morhenn et al., 2019).

Our analyses revealed that cardiac expression levels of *Creb1* and *F2rl2* remained unchanged across all our experimental models. Specifically, neither baseline characterization, nor evaluations following TAC or *CS1*-KO crossbreeding experiments, demonstrated any differential regulation of these genes. This consistency across diverse experimental settings suggests that miR-582 modulation does not significantly impact *Creb1* or *F2rl2* expression in the experimental cardiomyopathy models described above, underscoring the intricate, context-dependent nature of miRNA-mediated regulatory networks. In this regard, it should be noted that, to date no study has manipulated miR-582 in the murine heart *in vivo*, leaving its contribution to MI as well as its influence on *Creb1* and *F2rl2* regulation during MI development and progression unresolved.

To further investigate potential targets of miR-582-5p in the context of cardiac hypertrophy, we conducted *in silico* target predictions using four established algorithms. From the overlapping results, we selected seven genes with putative relevance to cardiac function. However, none of these genes exhibited differential expression in either the knockout or overexpression models—under basal conditions, following TAC surgery nor in *CS1*-KO crossbred animals. These findings suggest that miR-582-5p does not affect the transcriptional regulation of these predicted targets in the heart under the tested conditions. It is important to note, however, that our analysis was not exhaustive and additional targets likely exist, including confirmed targets in non-cardiac tissues, particularly in oncology.

In conclusion, our findings demonstrate that miR-582 modulation does not produce a discernible cardiac phenotype in the models described above, neither under basal conditions nor in response to pressure overload or genetic cardiomyopathy. However, this does not preclude a role for miR-582 in other cardiac settings, including forms of cardiomyopathy or heart diseases beyond those investigated here. Our experiments, conducted 2 weeks post-TAC, may not capture the full spectrum of miR-582’s influence. Alternative cardiomyopathy models, such as angiotensin II infusion, might reveal effects of miR-582 modulation on early stages of cardiac remodeling or effects of miR-582 modulation might only become apparent at even later remodeling stages or during aging. It is also conceivable that compensatory mechanisms by other genes or miRNAs mask the impact of miR-582 loss, while overexpression triggers counter‐regulatory defenses such as miRNA sponging or adjustments in related signaling pathways. Moreover, preliminary evidence suggests a potential involvement of miR-582 in MI, a condition not addressed in the present study. Nevertheless, our models already cover a wide range of potential effects, including acquired and genetic models of heart failure, experimentally manipulated by both, overexpression and downregulation of miR-582 *in vivo*.

Overall negative findings, as reported here, hold significant value for future research efforts. Making them accessible to the research community can prevent redundant studies and improve animal welfare by minimizing the unnecessary use of experimental animals. Our results may ultimately save time and resources by redirecting research efforts toward miRNAs or genes with greater impact in cardiomyopathy. Furthermore, our mouse models remain a useful resource for future *in vivo* studies exploring the role of miR-582, for example in the context of MI as well as non-cardiac diseases.

## Data Availability

The raw data supporting the conclusions of this article will be made available by the authors, without undue reservation.
